# Supplementary material for: Content-rich biological network constructed by mining PubMed abstracts
Source: BMC Bioinformatics. 2004 Oct 8;5:147. doi: 10.1186/1471-2105-5-147 (PMC528731; doi:10.1186/1471-2105-5-147)
Supplement: Additional File 2 — The original results of the above study (non-essential files are deleted to keep the file size under the limit set by BMC bioinformatics). [file 1471-2105-5-147-S2.bz2 › chilibotAdditionalFile2/dip05/390ID8700872E9868/html/VEGFC_FLT4.html]

 


 **VEGFC** and **FLT4** 
  
Found 127 abstracts in PubMed, retrieved 05.  
 

 What does Google say? 
 PDF only 
| .edu only 

---

**Interactive relationship** (e.g. stimulation, inhibition, etc)

**Stimulatory relationship**- Interestingly, VEGF C  [ **VEGFC** ]  stimulation of LECs induced also the formation of VEGFR 3  [ **FLT4** ]  VEGFR 2 heterodimers, in which VEGFR 3  [ **FLT4** ]  was phosphorylated only at three of the five sites while the two most C terminal tyrosine residues appeared not to be accessible for the VEGFR 2 kinase.  Ref: 12881528 J Biol ChemJ Biol Chem,
**Neutral relationship**- They belong to the larger family which also includes VEGF, placenta growth factor PlGF and VEGF B, VEGF C  [ **VEGFC** ]  and VEGF D are ligands for the endothelial cell specific tyrosine kinase receptors VEGFR 2 and VEGFR 3  [ **FLT4** ] .  Ref: 12888864 Thromb Haemost, 2003

**Non-interactive relationship** (e.g. studied together, co-existance, homology, etc.)

- OBJECTIVE To investigate the relationship between vascular endothelial growth factor C  [ **VEGFC** ]  VEGF C  [ **VEGFC** ]  expression, VEGFR 3  [ **FLT4** ]  expression, lymphangiogenesis and angiogenesis in human non small cell lung cancer NSCLC.  Ref: 12875690 Chin Med J (Engl), 2003
- RESULTS Of the 76 NSCLC cases, 55 were VEGF C  [ **VEGFC** ]  positive and 40 were VEGFR 3  [ **FLT4** ]  positive in cancer cells.  Ref: 12875690 Chin Med J (Engl), 2003
- Clinical significance of co expression of VEGF C  [ **VEGFC** ]  and VEGFR 3  [ **FLT4** ]  in non small cell lung cancer.  Ref: 12875690 Chin Med J (Engl), 2003
- The VEGFR 3  [ **FLT4** ]  specific mutant form of VEGF C  [ **VEGFC** ]  called VEGF C156S lacks blood vascular side effects but is sufficient for therapeutic lymphangiogenesis in a mouse model of lymphedema.  Ref: 12888864 Thromb Haemost, 2003
- Examination of the VEGF C  [ **VEGFC** ]  specific receptors VEGFR 2 KDR Flk 1 and VEGFR 3  [ **FLT4** ]  Flt 4  [ **FLT4** ]  demonstrated intense endothelial immunoreactivity for VEGFR 2, as well as VEGFR 2 and 3 expression on the majority of neoplastic cells, suggesting a possible role in autocrine paracrine neoplastic growth regulation.  Ref: 12861060 Mod Pathol, 2003
- The expression and levels of the VEGF C  [ **VEGFC** ]  and VEGF D cytokines, the VEGF receptors VEGFR 2 and VEGFR 3  [ **FLT4** ] , and newly described lymphatic endothelial markers, LYVE 1, Prox 1, podoplanin and 5 nucleotidase were assessed.  Ref: 12851706 Int J Oncol, 2003
- METHODS Seventy six NSCLC samples were stained for VEGF C  [ **VEGFC** ] , VEGFR 3  [ **FLT4** ]  and CD34 with immunohistochemical methods.  Ref: 12875690 Chin Med J (Engl), 2003
- VEGF C  [ **VEGFC** ]  expression and VEGFR 3  [ **FLT4** ]  expression were positively associated with lymph node metastasis and lymphatic invasion P <.  Ref: 12875690 Chin Med J (Engl), 2003
- CONCLUSIONS In NSCLC, VEGF C  [ **VEGFC** ]  and VEGFR 3  [ **FLT4** ]  are related to the lymphangiogenesis, angiogenesis, and occurrence and development of lung cancers.  Ref: 12875690 Chin Med J (Engl), 2003
- A significant positive correlation was found between VEGF C  [ **VEGFC** ]  expression and VEGFR 3  [ **FLT4** ]  expression in cancer cells P <.  Ref: 12875690 Chin Med J (Engl), 2003
